# Correction: Integrative bioinformatics approaches to map key biological markers and therapeutic drugs in Extramammary Paget’s disease of the scrotum

**DOI:** 10.1371/journal.pone.0259408

**Published:** 2021-10-27

**Authors:** Fatima Noor, Muhammad Hamzah Saleem, Jen-Tsung Chen, Muhammad Rizwan Javed, Wafa Abdullah Al-Megrin, Sidra Aslam

The following information is missing from the Acknowledgments: This research was funded by the Deanship of Scientific Research at Princess Nourah bint Abdulrahman University through the Fast-Track research funding program.

This information is also missing from the Funding statement. The correct Funding statement is as follows: This research was funded by the Deanship of Scientific Research at Princess Nourah bint Abdulrahman University through the Fast-Track research funding program.
